# Supplementary material for: Presence of Drug Interaction Between Penicillin and Hormonal Contraceptives in Women: A Scoping Review
Source: Healthcare (Basel). 2025 Jun 6;13(12):1364. doi: 10.3390/healthcare13121364 (PMC12193129; doi:10.3390/healthcare13121364)
Supplement: Supplementary file 1 [file healthcare-13-01364-s001.zip › healthcare-3607231-supplementary.pdf]

Search strategy

Presence of drug interaction between penicillin and hormonal contraceptives in women: a scoping review

1. Pubmed/Medline

#1 "Penicillins"[Mesh] OR (Antibiotics, Penicillin) OR (Penicillin Antibiotics) OR (Penicillin)

#2 "Contraceptive Agents"[Mesh] OR (Agents, Contraceptive) OR (Contraceptives) OR (Contraceptive) OR "Contraceptive Agents, Female"[Mesh] OR (Agents, Female Contraceptive) OR (Female Contraceptive Agents) OR (Contraceptives, Female) OR (Female Contraceptives) OR "Contraceptive Agents, Hormonal"[Mesh] OR (Hormonal Contraceptive Agents) OR (Contraceptive Agents, Female Hormonal) OR (Contraceptive Agents, Male Hormonal) OR "Contraceptives, Oral, Hormonal"[Mesh] OR (Contraceptive Agents, Oral, Hormonal) OR (Hormonal Oral Contraceptive Agent) OR (Oral Contraceptive Agents, Hormonal) OR (Hormonal Oral Contraceptive) OR (Contraceptive, Hormonal Oral) OR (Oral Contraceptive, Hormonal) OR (Oral Contraceptives, Hormonal) OR (Contraceptives, Hormonal Oral) OR (Hormonal Oral Contraceptives) OR (Hormonal Oral Contraceptive Agents) OR (Contraceptive Agents, Estrogen) OR (Estrogen Contraceptive Agents)

#1 AND #2

("Penicillins"[MeSH Terms] OR ("Penicillins"[Supplementary Concept] OR "Penicillins"[All Fields] OR "antibiotics penicillin"[All Fields] OR "Penicillins"[MeSH Terms] OR ("antibiotics"[All Fields] AND "penicillin"[All Fields])) OR ("Penicillins"[Supplementary Concept] OR "Penicillins"[All Fields] OR "penicillin antibiotics"[All Fields] OR "Penicillins"[MeSH Terms] OR ("penicillin"[All Fields] AND "antibiotics"[All

Fields])) OR ("benzylpenicillins"[All Fields] OR "penicillin g"[Supplementary Concept] OR "penicillin g"[All Fields] OR "benzylpenicillin"[All Fields] OR "penicillin g"[MeSH Terms] OR "penicilline"[All Fields] OR "penicillines"[All Fields] OR "Penicillins"[Supplementary Concept] OR "Penicillins"[All Fields] OR "penicillin"[All Fields] OR "Penicillins"[MeSH Terms])) AND ("Contraceptive Agents"[MeSH Terms] OR ("Contraceptive Agents"[Pharmacological Action] OR "Contraceptive Agents"[Supplementary Concept] OR "Contraceptive Agents"[All Fields] OR "agents contraceptive"[All Fields] OR "Contraceptive Agents"[MeSH Terms] OR ("contraceptive"[All Fields] AND "agents"[All Fields]) OR ("agents"[All Fields] AND "contraceptive"[All Fields])) OR ("contracept"[All Fields] OR "contracepted"[All Fields] OR "contracepting"[All Fields] OR "contraception"[MeSH Terms] OR "contraception"[All Fields] OR "contraceptions"[All Fields] OR "Contraceptive Agents"[Pharmacological Action] OR "Contraceptive Agents"[Supplementary Concept] OR "Contraceptive Agents"[All Fields] OR "contraceptive"[All Fields] OR "contraceptive devices"[MeSH Terms] OR ("contraceptive"[All Fields] AND "devices"[All Fields]) OR "contraceptive devices"[All Fields] OR "Contraceptive Agents"[MeSH Terms] OR ("contraceptive"[All Fields] AND "agents"[All Fields]) OR "contraceptives"[All Fields] OR "contraceptive s"[All Fields] OR "contraceptively"[All Fields]) OR ("contracept"[All Fields] OR "contracepted"[All Fields] OR "contracepting"[All Fields] OR "contraception"[MeSH Terms] OR "contraception"[All Fields] OR "contraceptions"[All Fields] OR "Contraceptive Agents"[Pharmacological Action] OR "Contraceptive Agents"[Supplementary Concept] OR "Contraceptive Agents"[All Fields] OR "contraceptive"[All Fields] OR "contraceptive devices"[MeSH Terms] OR ("contraceptive"[All Fields] AND "devices"[All Fields]) OR "contraceptive devices"[All Fields] OR "Contraceptive Agents"[MeSH Terms] OR ("contraceptive"[All Fields] AND "agents"[All Fields]) OR "contraceptives"[All Fields] OR "contraceptive s"[All Fields] OR "contraceptively"[All Fields]) OR "contraceptive agents, female"[MeSH Terms] OR ("contraceptive agents female"[Pharmacological Action] OR "contraceptive agents female"[Supplementary Concept] OR "contraceptive agents female"[All Fields] OR "contraceptive agents, female"[MeSH Terms] OR ("contraceptive"[All Fields] AND "agents"[All Fields] AND "female"[All Fields]) OR "female contraceptive agents"[All Fields] OR ("agents"[All Fields] AND "female"[All Fields] AND "contraceptive"[All Fields])) OR ("contraceptive agents female"[Pharmacological Action] OR "contraceptive agents female"[Supplementary

Concept] OR "contraceptive agents female"[All Fields] OR "female contraceptive agents"[All Fields] OR "contraceptive agents, female"[MeSH Terms] OR ("contraceptive"[All Fields] AND "agents"[All Fields] AND "female"[All Fields]) OR ("female"[All Fields] AND "contraceptive"[All Fields] AND "agents"[All Fields])) OR ("contraceptive agents female"[Pharmacological Action] OR "contraceptive agents female"[Supplementary Concept] OR "contraceptive agents female"[All Fields] OR "contraceptives female"[All Fields] OR "contraceptive agents, female"[MeSH Terms] OR ("contraceptive"[All Fields] AND "agents"[All Fields] AND "female"[All Fields]) OR "female contraceptive agents"[All Fields] OR ("contraceptives"[All Fields] AND "female"[All Fields])) OR ("contraceptive agents female"[Pharmacological Action] OR "contraceptive agents female"[Supplementary Concept] OR "contraceptive agents female"[All Fields] OR "female contraceptives"[All Fields] OR "contraceptive agents, female"[MeSH Terms] OR ("contraceptive"[All Fields] AND "agents"[All Fields] AND "female"[All Fields]) OR "female contraceptive agents"[All Fields] OR ("female"[All Fields] AND "contraceptives"[All Fields])) OR "contraceptive agents, hormonal"[MeSH Terms] OR ("contraceptive agents hormonal"[Pharmacological Action] OR "contraceptive agents hormonal"[Supplementary Concept] OR "contraceptive agents hormonal"[All Fields] OR "hormonal contraceptive agents"[All Fields] OR "contraceptive agents, hormonal"[MeSH Terms] OR ("contraceptive"[All Fields] AND "agents"[All Fields] AND "hormonal"[All Fields]) OR ("hormonal"[All Fields] AND "contraceptive"[All Fields] AND "agents"[All Fields])) OR ("contraceptive agents hormonal"[Pharmacological Action] OR "contraceptive agents hormonal"[Supplementary Concept] OR "contraceptive agents hormonal"[All Fields] OR "contraceptive agents, hormonal"[MeSH Terms] OR ("contraceptive"[All Fields] AND "agents"[All Fields] AND "hormonal"[All Fields]) OR "hormonal contraceptive agents"[All Fields] OR ("contraceptive"[All Fields] AND "agents"[All Fields] AND "female"[All Fields] AND "hormonal"[All Fields])) OR ("contraceptive agents hormonal"[Pharmacological Action] OR "contraceptive agents hormonal"[Supplementary Concept] OR "contraceptive agents hormonal"[All Fields] OR "contraceptive agents, hormonal"[MeSH Terms] OR ("contraceptive"[All Fields] AND "agents"[All Fields] AND "hormonal"[All Fields]) OR "hormonal contraceptive agents"[All Fields] OR ("contraceptive"[All Fields] AND "agents"[All Fields] AND "male"[All Fields] AND "hormonal"[All Fields])) OR "contraceptives, oral, hormonal"[MeSH Terms] OR ("contraceptives oral hormonal"[Pharmacological Action] OR "contraceptives oral

hormonal"[Supplementary Concept] OR "contraceptives oral hormonal"[All Fields] OR "contraceptive agents oral hormonal"[All Fields] OR "contraceptives, oral, hormonal"[MeSH Terms] OR ("contraceptives"[All Fields] AND "oral"[All Fields] AND "hormonal"[All Fields]) OR "hormonal oral contraceptives"[All Fields] OR ("contraceptive"[All Fields] AND "agents"[All Fields] AND "oral"[All Fields] AND "hormonal"[All Fields])) OR ("contraceptives oral hormonal"[Pharmacological Action] OR "contraceptives oral hormonal"[Supplementary Concept] OR "contraceptives oral hormonal"[All Fields] OR "contraceptives, oral, hormonal"[MeSH Terms] OR ("contraceptives"[All Fields] AND "oral"[All Fields] AND "hormonal"[All Fields]) OR "hormonal oral contraceptives"[All Fields] OR ("hormonal"[All Fields] AND "oral"[All Fields] AND "contraceptive"[All Fields] AND "agent"[All Fields])) OR ("contraceptives oral hormonal"[Pharmacological Action] OR "contraceptives oral hormonal"[Supplementary Concept] OR "contraceptives oral hormonal"[All Fields] OR "contraceptives, oral, hormonal"[MeSH Terms] OR ("contraceptives"[All Fields] AND "oral"[All Fields] AND "hormonal"[All Fields]) OR "hormonal oral contraceptives"[All Fields] OR ("oral"[All Fields] AND "contraceptive"[All Fields] AND "agents"[All Fields] AND "hormonal"[All Fields])) OR ("contraceptives oral hormonal"[Pharmacological Action] OR "contraceptives oral hormonal"[Supplementary Concept] OR "contraceptives oral hormonal"[All Fields] OR "hormonal oral contraceptive"[All Fields] OR "contraceptives, oral, hormonal"[MeSH Terms] OR ("contraceptives"[All Fields] AND "oral"[All Fields] AND "hormonal"[All Fields]) OR "hormonal oral contraceptives"[All Fields] OR ("hormonal"[All Fields] AND "oral"[All Fields] AND "contraceptive"[All Fields])) OR ("contraceptives oral hormonal"[Pharmacological Action] OR "contraceptives oral hormonal"[Supplementary Concept] OR "contraceptives oral hormonal"[All Fields] OR "contraceptives, oral, hormonal"[MeSH Terms] OR ("contraceptives"[All Fields] AND "oral"[All Fields] AND "hormonal"[All Fields]) OR "hormonal oral contraceptives"[All Fields] OR ("contraceptive"[All Fields] AND "hormonal"[All Fields] AND "oral"[All Fields])) OR ("contraceptives oral hormonal"[Pharmacological Action] OR "contraceptives oral hormonal"[Supplementary Concept] OR "contraceptives oral hormonal"[All Fields] OR "oral contraceptive hormonal"[All Fields] OR "contraceptives, oral, hormonal"[MeSH Terms] OR ("contraceptives"[All Fields] AND "oral"[All Fields] AND "hormonal"[All Fields]) OR "hormonal oral contraceptives"[All Fields] OR ("oral"[All Fields] AND "contraceptive"[All Fields] AND "hormonal"[All Fields])) OR

("contraceptives oral hormonal"[Pharmacological Action] OR "contraceptives oral hormonal"[Supplementary Concept] OR "contraceptives oral hormonal"[All Fields] OR "oral contraceptives hormonal"[All Fields] OR "contraceptives, oral, hormonal"[MeSH Terms] OR ("contraceptives"[All Fields] AND "oral"[All Fields] AND "hormonal"[All Fields]) OR "hormonal oral contraceptives"[All Fields] OR ("oral"[All Fields] AND "contraceptives"[All Fields] AND "hormonal"[All Fields])) OR ("contraceptives oral hormonal"[Pharmacological Action] OR "contraceptives oral hormonal"[Supplementary Concept] OR "contraceptives oral hormonal"[All Fields] OR "contraceptives, oral, hormonal"[MeSH Terms] OR ("contraceptives"[All Fields] AND "oral"[All Fields] AND "hormonal"[All Fields]) OR "hormonal oral contraceptives"[All Fields] OR ("contraceptives"[All Fields] AND "hormonal"[All Fields] AND "oral"[All Fields])) OR ("contraceptives oral hormonal"[Pharmacological Action] OR "contraceptives oral hormonal"[Supplementary Concept] OR "contraceptives oral hormonal"[All Fields] OR "hormonal oral contraceptives"[All Fields] OR "contraceptives, oral, hormonal"[MeSH Terms] OR ("contraceptives"[All Fields] AND "oral"[All Fields] AND "hormonal"[All Fields]) OR ("hormonal"[All Fields] AND "oral"[All Fields] AND "contraceptives"[All Fields])) OR ("contraceptives oral hormonal"[Pharmacological Action] OR "contraceptives oral hormonal"[Supplementary Concept] OR "contraceptives oral hormonal"[All Fields] OR "hormonal oral contraceptive agents"[All Fields] OR "contraceptives, oral, hormonal"[MeSH Terms] OR ("contraceptives"[All Fields] AND "oral"[All Fields] AND "hormonal"[All Fields]) OR "hormonal oral contraceptives"[All Fields] OR ("hormonal"[All Fields] AND "oral"[All Fields] AND "contraceptive"[All Fields] AND "agents"[All Fields])) OR ("contraceptives oral hormonal"[Pharmacological Action] OR "contraceptives oral hormonal"[Supplementary Concept] OR "contraceptives oral hormonal"[All Fields] OR "contraceptive agents estrogen"[All Fields] OR "contraceptives, oral, hormonal"[MeSH Terms] OR ("contraceptives"[All Fields] AND "oral"[All Fields] AND "hormonal"[All Fields]) OR "hormonal oral contraceptives"[All Fields] OR ("contraceptive"[All Fields] AND "agents"[All Fields] AND "estrogen"[All Fields])) OR ("contraceptives oral hormonal"[Pharmacological Action] OR "contraceptives oral hormonal"[Supplementary Concept] OR "contraceptives oral hormonal"[All Fields] OR "contraceptives, oral, hormonal"[MeSH Terms] OR

("contraceptives"[All Fields] AND "oral"[All Fields] AND "hormonal"[All Fields]) OR "hormonal oral contraceptives"[All Fields] OR ("estrogen"[All Fields] AND "contraceptive"[All Fields] AND "agents"[All Fields])))

2. EMBASE

#1 AND #4

#2 OR #3

'contraceptive agents, hormonal'/exp OR 'contraceptive agents, hormonal'

'contraceptive agent'/exp

'penicillins'/exp OR penicillins

3. Web of Science

Penicillins and (Contraceptive Agents) OR (Contraceptive Agents Female) OR (Contraceptive Agents Hormonal) OR (Contraceptives Oral Hormonal)

4. Virtual Health Library

#1 "Penicilinas" OR "Penicillins"

#2 "Anticoncepcionais" OR "Contraceptive Agents" OR "Anticonceptivos" OR "Anticoncepcionais Orais" OR "Contraceptives, Oral" OR "Anticonceptivos Orales" OR "Contraceptivos Hormonais" OR "Contraceptive Agents, Hormonal" OR "Agentes Anticonceptivos Hormonales"

Preferred Reporting Items for Systematic reviews and Meta-Analyses extension for Scoping Reviews (PRISMA-ScR) Checklist

SECTION	ITEM	PRISMA-ScR CHECKLIST ITEM	REPORTED ON PAGE #
**TITLE**			
Title	1	Identify the report as a scoping review.	1
**ABSTRACT**			
Structured summary	2	Provide a structured summary that includes (as applicable): background, objectives, eligibility criteria, sources of evidence, charting methods, results, and conclusions that relate to the review questions and objectives.	1-2
**INTRODUCTION**			
Rationale	3	Describe the rationale for the review in the context of what is already known. Explain why the review questions/objectives lend themselves to a scoping review approach.	2-3
Objectives	4	Provide an explicit statement of the questions and objectives being addressed with reference to their key elements (e.g., population or participants, concepts, and context) or other relevant key elements used to conceptualize the review questions and/or objectives.	3
**METHODS**			
Protocol and registration	5	Indicate whether a review protocol exists; state if and where it can be accessed (e.g., a Web address); and if available, provide registration information, including the registration number.	4
Eligibility criteria	6	Specify characteristics of the sources of evidence used as eligibility criteria (e.g., years considered, language, and publication status), and provide a rationale.	4
Information sources*	7	Describe all information sources in the search (e.g., databases with dates of coverage and contact with authors to identify additional sources), as well as the date the most recent search was executed.	4
Search	8	Present the full electronic search strategy for at least 1 database, including any limits used, such that it could be repeated.	Additional file 3
Selection of sources of evidence†	9	State the process for selecting sources of evidence (i.e., screening and eligibility) included in the scoping review.	4
Data charting process‡	10	Describe the methods of charting data from the included sources of evidence (e.g., calibrated forms or forms that have been tested by the team before their use, and whether data charting was done independently or in duplicate) and any processes for obtaining and confirming data from investigators.	5
Data items	11	List and define all variables for which data were sought and any assumptions and simplifications made.	5

SECTION	ITEM	PRISMA-ScR CHECKLIST ITEM	REPORTED ON PAGE #
Critical appraisal of individual sources of evidence§	12	If done, provide a rationale for conducting a critical appraisal of included sources of evidence; describe the methods used and how this information was used in any data synthesis (if appropriate).	NA
Synthesis of results	13	Describe the methods of handling and summarizing the data that were charted.	5
**RESULTS**			
Selection of sources of evidence	14	Give numbers of sources of evidence screened, assessed for eligibility, and included in the review, with reasons for exclusions at each stage, ideally using a flow diagram.	6
Characteristics of sources of evidence	15	For each source of evidence, present characteristics for which data were charted and provide the citations.	7-11; 20-25
Critical appraisal within sources of evidence	16	If done, present data on critical appraisal of included sources of evidence (see item 12).	NA
Results of individual sources of evidence	17	For each included source of evidence, present the relevant data that were charted that relate to the review questions and objectives.	7-11; 20-25
Synthesis of results	18	Summarize and/or present the charting results as they relate to the review questions and objectives.	7-11
**DISCUSSION**			
Summary of evidence	19	Summarize the main results (including an overview of concepts, themes, and types of evidence available), link to the review questions and objectives, and consider the relevance to key groups.	11-12
Limitations	20	Discuss the limitations of the scoping review process.	12
Conclusions	21	Provide a general interpretation of the results with respect to the review questions and objectives, as well as potential implications and/or next steps.	14
**FUNDING**			
Funding	22	Describe sources of funding for the included sources of evidence, as well as sources of funding for the scoping review. Describe the role of the funders of the scoping review.	15

JBI = Joanna Briggs Institute; PRISMA-ScR = Preferred Reporting Items for Systematic reviews and Meta-Analyses extension for Scoping Reviews.
* Where *sources of evidence* (see second footnote) are compiled from, such as bibliographic databases, social media platforms, and Web sites.
† A more inclusive/heterogeneous term used to account for the different types of evidence or data sources (e.g., quantitative and/or qualitative research, expert opinion, and policy documents) that may be eligible in a scoping review as opposed to only studies. This is not to be confused with *information sources* (see first footnote).
‡ The frameworks by Arksey and O'Malley (6) and Levac and colleagues (7) and the JBI guidance (4, 5) refer to the process of data extraction in a scoping review as data charting.
§ The process of systematically examining research evidence to assess its validity, results, and relevance before using it to inform a decision. This term is used for items 12 and 19 instead of "risk of bias" (which is more applicable to systematic reviews of interventions) to include and acknowledge the various sources of evidence that may be used in a scoping review (e.g., quantitative and/or qualitative research, expert opinion, and policy document).

From: Tricco AC, Lillie E, Zarin W, O'Brien KK, Colquhoun H, Levac D, et al. PRISMA Extension for Scoping Reviews (PRISMAScR): Checklist and Explanation. Ann Intern Med. 2018;169:467–473. doi: 10.7326/M18-0850.
